# Evaluation of a menu box delivery service for Australian long-day care services to improve food provision and child intake: a cluster randomised controlled trial

**DOI:** 10.1017/S1368980023002136

**Published:** 2023-12

**Authors:** Shabnam Kashef, Lucinda K Bell, Victoria Brown, Claire Gardner, Dorota Zarnowiecki, Samantha Morgillo, Jennifer C Arguelles, David N Cox, Rebecca K Golley

**Affiliations:** 1 Caring Futures Institute, College of Nursing and Health Sciences, Flinders University, Bedford Park South Australia, GPO Box 2100, Adelaide, SA 5001, Australia; 2 Deakin Health Economics, Institute for Health Transformation, Deakin University, Geelong, VIC, Australia; 3 Nutrition Australia Victoria Division, Docklands, VIC, Australia; 4 Commonwealth Scientific and Industrial Research Organisation (CSIRO) Health and Biosecurity, Adelaide, SA, Australia

**Keywords:** Preschool, Childcare, Children, Diet, Food environments, Public health

## Abstract

**Objective:**

To evaluate the impact of a menu box delivery service tailored to the long-day care (LDC) setting on improving menu compliance with recommendations, children’s diet quality and dietary intake while in care.

**Design:**

A cluster randomised controlled trial in LDC centres randomly assigned to an intervention (menu box delivery) or comparison (menu planning training) group. The primary outcome was child food provision and dietary intake. Secondary outcomes include menu compliance and process evaluation, including acceptability, fidelity and menu cost (per child, per day).

**Setting:**

South Australian LDC centres.

**Participants:**

Eight LDC centres (*n* 224 children) provided data.

**Results:**

No differences were observed in serves/d between intervention and comparison centres, for provision (intervention, 0·9 inter-quartile range (IQR) 0·7–1·2; comparison, 0·8 IQR 0·5–1·3) or consumption (intervention, 0·5 IQR 0·2–0·8; comparison, 0·5 IQR 0·3–0·9) of vegetables. Child food provision and dietary intake were similar across both groups for all food groups (*P* < 0·05). At follow-up, all intervention centres met menu planning guidelines for vegetables, whereas only one comparison centre met guidelines. Intervention centre directors found the menu box delivery more acceptable than cooks. Cost of the intervention was AUD$2·34 greater than comparison centres (intervention, AUD$4·62 (95 % CI ($4·58, $4·67)); comparison, AUD$2·28 (95 % CI ($2·27, $2·30)) per child, per day).

**Conclusions:**

Menu compliance can be improved via a menu delivery service, delivering equivalent impacts on child food provision and dietary intake compared with an online training programme. Further exploration of cooks acceptability and cost is essential before scaling up to implementation.

A nutritious diet supports health, growth and development. Diet is a modifiable risk factor for chronic disease and can prevent the burden of disease later in life. Early intervention is key to developing healthy habits, especially as behaviours developed in childhood often carry through to adolescence and adulthood^(1–3)^. In Australia and internationally, dietary guidelines recommend the types and amounts of foods for optimal health, growth and development^(4,5)^. However, the dietary patterns of both children and adults do not meet recommendations^(6–8)^. For example, only 6 % of Australian children aged 2–17 years eat the recommended number of vegetables^(8,9)^. Community settings where children spend time and consume food, such as early childhood education and care (ECEC), have been identified as key spaces to target dietary behaviours^(10,11)^.

In Australia, over half of children under 5 years old attend ECEC services^(12)^. The most common setting for formal childcare in Australia is long-day care (LDC). Food is often provided onsite in LDC centres, typically prepared by a cook, e.g. 70 % of LDC centres in South Australia (Unpublished, Egan and Cox, 2015). Centres operate for around 8 h/d, which means children can consume between 40 and 60 % of their daily food intake while in care^(13,14)^.

Within Australian LDC centres that provide meals onsite, centre cooks are typically responsible for menu planning, food purchasing and food preparation. While centre cooks are not required to undertake formal nutrition training, they are expected to provide a healthy, nutritious menu to children in care^(15)^. Nutrition policy and menu planning guidelines, such as the Caring for Children^(16)^ resource and the Victorian Menu planning guidelines for LDC^(17)^, are available to cooks and underpin a range of programmes to support cooks’ nutrition knowledge and skills to plan and provide appropriate meals. Specifically, such guidelines outline the appropriate number of serves from each food group that should be provided to children over each eating occasion throughout the day. Each day in care should provide children with about half of their recommended daily intake from each of the five food groups (vegetables and legumes; fruit; cereals and breads; dairy and alternatives; meat and alternatives). Similar policies internationally include the Voluntary Food and Drink Guidelines for Early Years Settings in England^(18)^ and the Caring for Our Children: National Health and Safety Performance Standards in the United States of America^(19)^, which recommend centre food provision to meet the requirements outlined by the Child and Adult Care Food Program.

Analysis of childcare menus both in Australia and internationally shows that centres typically do not meet menu guidelines, particularly for vegetables^(20–22)^. Previous studies have reported numerous barriers that impede implementation of or compliance with guidelines in the childcare setting^(23–25)^. This includes insufficient menu planning tools and resources, a lack of time or nutrition knowledge, a lack of awareness of dietary guidelines and a lack of confidence^(25)^. For example, interviews with LDC staff indicate that cooks, directors and educators rely on personal knowledge or online research to determine the nutritional adequacy of foods provided to children in care, rather than using evidence-based resources^(26)^. These barriers are further exacerbated when paired with staff beliefs around the perception that healthy foods such as vegetables will cost more and may not be liked by children, resulting in food waste^(27)^. This highlights the need for innovative approaches to tackle such barriers in these settings.

Meal kit subscription services have been growing in popularity across many countries including Australia and the United States of America^(28)^. Domestic models have been positively received by families worldwide as a solution to integrating home cooking into busy, time-poor lifestyles^(29,30)^. The meal kit subscription service food model delivers a package or pre-portioned amount of ingredients and recipes to homes of subscribers^(29–31)^. A novel food service model for LDC can pair the food supply to the centre menu to provide a meal kit subscription service compliant with sector menu guidelines. By providing a menu tailored to the number of children attending centres, recipe ingredients can be delivered in adequate quantities that align with menu planning guidelines. Such a model could address barriers to implementing guidelines across the centre menu and the supply chain (food procurement). Furthermore, this model has the potential to save centres time cost for labour for food provision and procurement. Additionally, the streamlined menu approach could allow for purchasing power to increase expenditure on ingredients previously deemed too expensive. To current knowledge, there is no evidence of literature describing the use of a meal kit-style food service model in the ECEC setting.

Therefore, the aim of this study was to evaluate the impact of a meal kit-style intervention tailored to the LDC setting, compared with standard practice, on child food provision and dietary intake of the five food groups (vegetables and legumes; fruit; cereals and breads; dairy and alternatives; meat and alternatives) for preschool children, particularly vegetables, while in LDC. The secondary aim of this study was to evaluate the impact of the intervention on menu compliance with menu planning guidelines. Centre cook, director acceptability and cost were also assessed.

## Methods

### Study design

A cluster randomised controlled trial design in South Australian LDC centres. Centres were randomly allocated to one of two study groups over the 12-week study period. Intervention centres received a weekly menu box delivery service to provide a predetermined menu compliant with the Victorian Menu Planning Guidelines, designed by dietitians experienced in the LDC setting for the purpose of the present study^(17)^. There are no current standardised menu guidelines for LDC settings in South Australia. For this reason, the Victorian Menu Planning Guidelines were used in this study.

Comparison centres were asked to utilise an online menu assessment tool and an online cook training tool to support cooks to develop and deliver a menu that was compliant with the Victorian Menu Planning Guidelines reflective of recommended practice in LDC^(17)^. The study has been described in detail in a previously published protocol^(32)^. The study protocol was prospectively registered within the Australian New Zealand Clinical Trials Registry (ACTRN12620000296932).

### Study participants

Study participants are reported in detail in the published protocol paper^(32)^. Briefly, to be eligible, LDC centres needed to be located in the metropolitan area of the city of Adelaide, have an on-site cook who made menu planning decisions and prepared and served a minimum of one main meal (lunch) and two mid-meal snacks (morning and afternoon) per day. Only private LDC centres were approached for this study as the ethics requirements to engaged public centres were lengthy and did not align with the study timeline^(32)^. Within centres, children aged between 2 and 5 years who were present on data collection days were eligible to participate. Children below the age of 2 years were excluded as menu planning guidelines, and centre menus typically differ across this age group. Furthermore, children under two require texturally modified meals that need to be developmentally appropriate for each individual child individual developmental trajectory. Therefore, this age group was excluded from this study^(17)^. Children with dietary requirements and allergies that prevented them from receiving the standard or vegetarian centre menu were excluded.

### Recruitment

As detailed in the protocol paper, eligible centres were recruited in partnership with a local childcare service provider and stratified by socio-economic status^(32,33)^. From this list, centres were invited to participate in random order until the required sample size of 180 children was achieved^(34)^. Sample size calculations were conducted using G * Power Software40 based on *α* of 0·05 and power of 0·80. Directors of invited centres were emailed an invitation to participate including study information followed by a phone call from the research team to confirm eligibility. Face-to-face meetings were organised with directors and cooks at centres that expressed interest to participate. Study participants in centres included centre directors, cooks, educators (graduate certificate qualification), teachers (teaching degree qualification) and children. Centre directors provided consent for the centre to be involved in the study. Cooks provided written informed consent for themselves. Parents of children attending the centre were provided information about the study and received an opportunity to opt their child out of data collection.

### Randomisation and group allocation

Centres were randomly allocated to either the intervention (menu box delivery) or comparison (menu planning) group after baseline data collection was complete. Centres were stratified into two equal groups matched for centre size (based on child attendance numbers) and centre socio-economic status using Socio-Economic Indexes for Areas^(33)^. These two groups were then randomly assigned by a staff member outside the study research team to receive the intervention or comparator using a random number generator (random.org).

### The menu box delivery intervention

Centres allocated to the intervention received a weekly menu box delivery service that included all ingredients and recipes required for morning snack, lunch and afternoon snack for the week, tailored to the number of children attending the centre. Intervention centres were provided with menu packs that included information about the delivery process, copies of tailored recipes and ingredient lists for each week of the 4-week menu. Ingredients were delivered weekly to centres by a local grocery supplier. The cost of the menu box delivery service, including ingredients, menus/recipes and delivery, was covered by the research team. Centres were asked to apply standard practices and policies to manage the preferences and dietary requirements of children in their care.

**Fig. 1:**
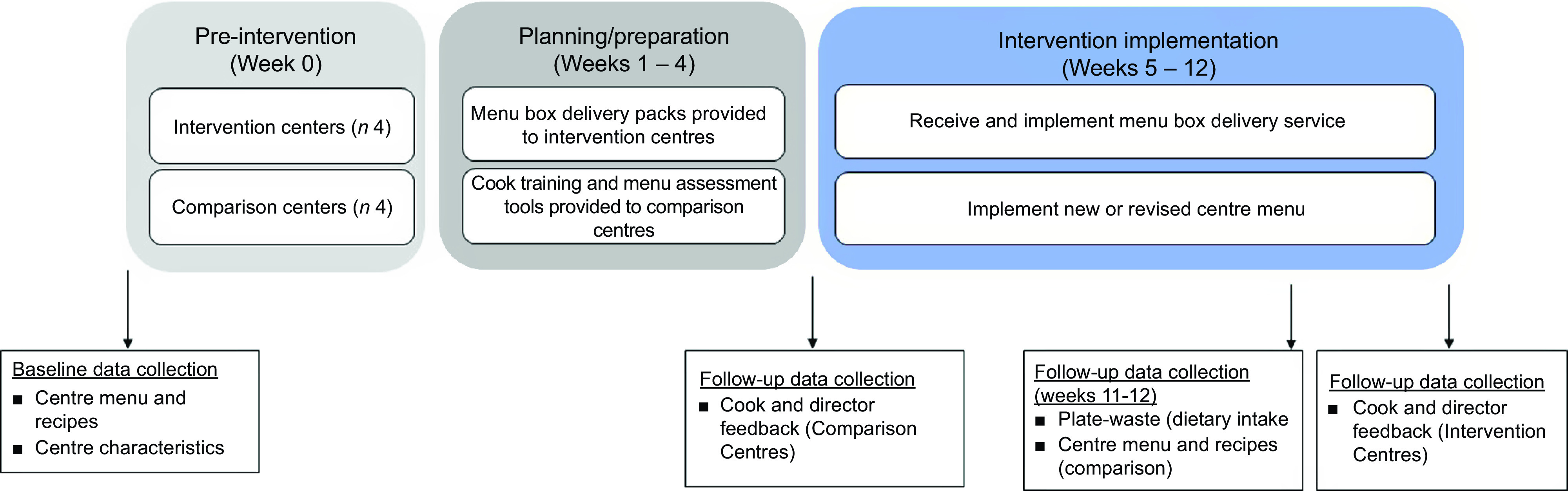
Intervention flow and data collection points.

Comparison centres were provided with a self-paced online menu planning training and an online menu assessment tool designed for LDC cooks to support the implementation of a centre menu that met Menu Planning Guidelines^(17)^. After completing the training, cooks implemented a new menu at their centre as per usual practice. The total intervention period was 12 weeks (October – December 2020), which included a 4-week menu planning period and an 8-week menu implementation period (Fig. 1).

### Data collection and entry

Data were collected on two occasions: (i) at baseline, pre-intervention between July and October 2020 and (ii) at follow-up, in weeks 11 and 12 of the intervention implementation period (between November and December 2020). The primary outcomes were child food provision and child dietary intake. Secondary outcomes were menu compliance and intervention acceptability by staff. Due to COVID-19 restrictions at the time, modifications were made to the collection of dietary data at the follow-up time point. Due to differences in methods at baseline and follow-up, only follow-up dietary data were analysed and compared between groups.

#### Centre, staff and child socio-demographic characteristics

At baseline, operational data for the centres were collected, including average attendance and nutrition policies. Baseline cook characteristics including age, experience, hours worked/week and relevant qualifications were collected. Age and gender were collected for all children involved in dietary data collection.

#### Primary outcomes: food provision and child dietary intake

The primary outcomes assessed were child food provision (what children were served at mealtimes) and child dietary intake assessed against the Victorian Menu Planning Guidelines for five food groups (vegetables and legumes; fruit; cereals and breads; dairy and alternatives and meat and alternatives) and discretionary food and drinks (foods and drinks that do not fit in the five food groups as they are nutrient poor and typically higher in kilojoules, saturated fat, added sugars and/or added salt) as classified in the Australian Dietary Guidelines^(17,35)^. Detailed information about the outcome measures are provided in the protocol paper^(32)^. Food provision, consumption and waste were measured at mealtimes, with a particular focus on vegetable provision and consumption.

Children’s food provision and intake were measured using the standardised weighed plate wastage method for lunch and two mid-meal snacks. To adapt to COVID-19 restrictions at follow-up data collection, measurement of plate waste was modified to incorporate a hybrid use of photography and weight measures, as researchers were not permitted to enter rooms during mealtimes^(36–38)^. At the start of the meal, educators were asked to serve three portions as they would for a child in the room, which were set aside for the researcher to weigh and photograph after meal service as a ‘reference portion’. Educators were then instructed to take photographs of child meals during mealtimes and any additional serves from a birds-eye view with a 30-cm ruler in the frame. At the end of the meal, educators collected all left-over bowls with remaining food waste intact, which were provided to the researcher situated outside the room to be weighed. Educators were also provided with a mealtime checklist to mark off how many serves of each food item each child was provided. One day of eating (morning tea, lunch and afternoon tea) per child was measured. Two trained dietitians digitally assessed each child’s meal photograph in comparison with the reference photos to estimate the amount of food in each photograph. The ruler in the images was used as a guide to compare plate waste images to images of the reference portion, to calculate provision. The amount of food was estimated both in grams (g) and as a percentage of the reference portions, in units of 10 %; for example, 80 % or 120 % (if more than the reference portion). Consumption was calculated by subtracting weighed waste from the estimated provision.

All foods were entered into Excel, and an eight-digit food code was assigned to individual food items (e.g. raw carrot) using the AUSNUT 2011–2013 database^(39)^. Food item and specific food code decisions were made by research staff and discussed and approved by the chief investigator. Ten percent of entries were double coded to ensure consistency between research staff. Recipes for mixed meals (e.g. curry) were provided by centre cooks and entered into FoodWorks Professional Version 10 (Xyris Software, Queensland, Australia) using a standard protocol developed for this study to determine the proportional weight of each ingredient according to total recipe weight by food group based on the eight-digit code assigned to each food item. The amount of food consumed (intake) was calculated by subtracting food waste (g) from food provision (g). All food items were then converted to standard servings of their respective food groups according to the Victorian Menu Planning Guidelines^(17)^. For example, one serve of vegetables and legumes is equivalent to 75 g of raw or cooked vegetables.

#### Secondary outcomes: menu compliance and intervention acceptability, fidelity and cost

Menu compliance was measured by assessing two consecutive weeks of centre menus against the Victorian Menu Planning Guidelines^(17)^. Centre cooks were asked to provide: (1) a copy of their full centre menu; (2) recipes for 2 weeks of the menu and (3) the number of children provided for. Recipes were assessed by a trained dietitian using the serve size and quantities outlined by the guidelines for each of the five food groups.

Cook and director feedback was obtained through structured interviewer-administered questionnaires to evaluate cooks’ acceptability of intervention components and to collect feedback on training material at follow-up. The purpose-designed questionnaires included four items for menu box delivery acceptability, four items for online cook training acceptability and four items for menu assessment tool acceptability for both directors and cooks (see Table 1). Items were rated using a five-point Likert scale from strongly disagree to strongly agree. Intervention centre cook questionnaires were administered at completion of the 8-week menu box delivery intervention, while the comparison centre cook questionnaire was administered following the training and menu revision phase (Fig. 1). Fidelity was measured by adherence to the menu box delivery service (intervention), reported by cooks; and completion of the online cook training and weeks of the menu assessed using the menu assessment tool (comparison). The cost of the menu was measured in Australian Dollars (2020) as cost per child per day. Cooks were asked to collect all menu invoices over the 8-week intervention period, and intervention centre invoices were provided directly from the supplier. An average menu cost per week was calculated then divided by average weekly attendance to determine the menu cost per child/d.

**Table 1:** Overall acceptability and satisfaction of the menu box delivery service, online cook training and menu assessment tool reported by centre cooks and directors at follow-up (=8)

Number of responders that agreed or strongly agreed with the following statements	Directors (*n* 4)	Cooks (*n* 4)
	*n*	
Intervention centres		
If able to, my centre would continue to use the menu box delivery service.	4	2
I noticed an improvement in vegetable intake of children who attended the service when using the menu box delivery service.	2	0
I believe the children at the centre, benefitted from the menu box delivery service.	4	2
I would recommend the menu box delivery service to other centres.	4	1
Number of responders that agreed or strongly agreed with the following statements	Directors (*n* 4)	Cooks (*n* 4)
Comparison centres: online cook training		
Using the online cook training is an acceptable training tool for cooks at this centre.	2	2
My centre would continue to use the online cook training to plan menus.	1	2
I would recommend the online cook training to other centres.	1	2
I believe the children at the centre, benefitted from the use of the online cook training.	1	1
Comparison centres: menu assessment tool		
Using the menu assessment tool is an acceptable method for assessing our services menu compliance against the dietary guidelines.	1	2
My centre would continue to use the menu assessment tool to plan menus.	1	1
I would recommend the menu assessment tool to other centres.	1	2
I believe the children at the centre, benefitted from the use of menu assessment tool.	1	1

### Statistical analysis

All statistical analyses were performed using SPSS 24.0 statistical software. Data were checked and cleaned prior to analyses and visually assessed for normality using frequency histograms, which were then compared with the results of Kolmogorov–Smirnov and Shapiro–Wilk tests of normality. As child food provision and intake data were not normally distributed, data are reported as median and inter-quartile range (IQR). Socio-demographic data are reported as frequency (%) or mean (sd) and intervention acceptability is reported as the number of respondents who agreed or strongly agreed with the acceptability statements.

The effect of the intervention on children’s food provision and intake was determined using a linear mixed-effects model, adjusting for clustering of centres (random effect) and controlling for child age and gender, socio-economic status of centre location and centre size (fixed effects). Only children with complete data for all measured eating occasions including lunch and morning and afternoon snacks were included in the analyses. Log-transformation was performed for variables that did not fit model assumptions. Estimates for transformed variables are reported as the ratio of geometric means, whereas non-transformed variables are reported as geometric means. Statistical significance was considered at *P* < 0·05. Bootstrapped means and 95 % CIs were calculated for menu cost per child per day.

## Results

### Sample characteristics

**Fig. 2:**
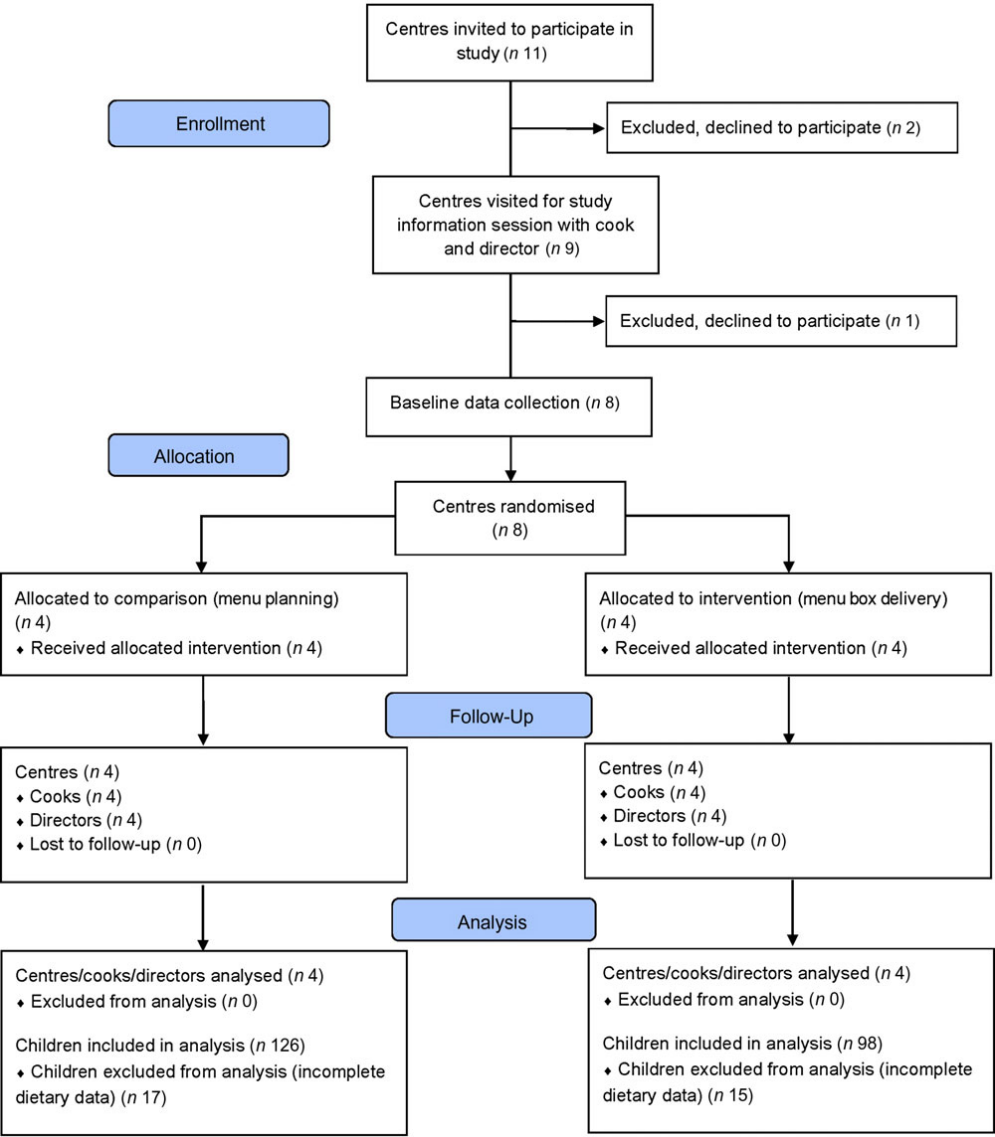
CONSORT flow diagram of centres and children through the study.

Eight of 11 LDC centres approached to participate in the study agreed to participate (four intervention, four comparison: Fig. 2). Reasons provided by centres that declined include staff changes (*n* 1) or a lack of time (*n* 2). Baseline demographic data were collected for centres (*n* 8) and cooks (*n* 8) and are presented in Table 2. At follow-up, dietary data were collected for 224 children (*n* 126 comparison centres, *n* 98 intervention centres with complete data). Child demographic characteristics are presented in Table 2. A total of eight cooks and eight directors completed follow-up acceptability questionnaires.

**Table 2:** Demographic characteristics of comparison and intervention; centres, centre cooks and children included in follow up data analysis*

Characteristics	All centres (*n* 8)		Comparison (*n* 4)		Intervention (*n* 4)	
	Mean	SD	Mean	SD	Mean	SD
Centre characteristics						
Centre size, approved places	102	16	102	19	103	21
Number of children attending each day	60	14	60	12	61	18
	*n* †		*n* †		*n* †	
Socioeconomic status of centre location						
Low socio-economic status	3		2		1	
Mid socio-economic status	2		1		1	
High socio-economic status	3		1		2	
Children of Aboriginal or Torres Strait Islander background enrolled at centre	12		4		8	
	Mean	sd	Mean	sd	Mean	sd
Cook characteristics (*n* 8)						
Cook age, years	33·1	6·6	33·5	3·0	32·8	7·7
	*n*		*n*		*n*	
Gender						
Female	8		4		4	
	Mean	sd	Mean	sd	Mean	sd
Years’ experience						
In current role	3·2	0·6	3·1	1·7	3·3	1·4
In childcare sector	8·9	1·8	8·3	6·3	9·5	12·4
As cook, all settings	5·5	1·1	3·9	1·7	7·1	4·7
	*n*		*n*		*n*	
Highest education/level of training						
Tertiary education (University)	0		0		0	
Certificate or diploma	7		3		4	
No formal qualifications (on the job training)	1		1		0	
	Mean	sd	Mean	sd	Mean	sd
Hours worked/week	26·8	5·4	27·5	2·9	26·1	5·0
Child characteristic (*n* 224)	All Children (*n* 224)		Comparison group (*n* 126)		Intervention group (*n* 98)	
	*n* ‡	%	*n* ‡	%	*n* ‡	%
Gender						
Female	96	43	55	44	41	42
Male	128	57	71	56	57	58
	Median	IQR	Median	IQR	Median	IQR
Age (months)	48	39–56	49	40–55	46	33–57
	*n*	%	*n*	%	*n*	%
Number of children by room/age group						
Toddler (2–3 years)	40	18	19	15	21	21
Pre-kindergarten (3–4 years)	80	36	51	41	29	30
Kindergarten (4–5 years)	104	46	56	44	48	49
Number of children by socio-economic status of centre location*						
Low socio-economic status	84	38	42	33	42	43
Mid socio-economic status	41	18	23	18	18	18
High socio-economic status	99	44	61	49	38	39
Number of children of Aboriginal or Torres Strait Islander background in sample	1	0	0	0	1	1

IQR, inter-quartile range.

* Data presented as *n* (%) or median (IQR).

† Three socio-economic status categories, low, mid and high, were formed using Socio-Economic Indexes for Areas determined by centre postcode. Indices of 1–3 were categorised as indicating ‘low’ SES, 4–7 as ‘mid’ and 8–10 as ‘high’^(33)^.

‡ *n* 3 missing in comparison centres (2·4 %).

### Child food provision and dietary intake

At follow-up, median (IQR) child daily vegetable provision within intervention centres was 0·9 (0·7–1·2) serves/d, compared with 0·8 (0·5–1·3) serves/d in the comparison centres (Table 3). This is equivalent to approximately 8·4 g greater vegetable provision for intervention centres (67·9 g/d, IQR 49·6–91·4) than comparison centres (59·5 g/d, IQR 35·0–95·3). Child daily vegetable intake was similar across the two groups, at 0·5 (0·2–0·8) serves/d in intervention centres and 0·5 (0·3–0·9) serves/d in comparison centres. There was no statistically significant difference in provision or intake for any food group between the intervention and comparison centres at follow-up (*P* > 0·05) (Table 4). Provision of fruit (intervention *v*. comparison centres; 0·6 (0·5–0·9) *v*. 1·5 (0·7–2·4) serves/d) and cereals and breads (0·9 (0·9–2·0) *v*. 2·0 (1·5–2·9) serves/d) were lower in intervention centres compared with comparison centres. Provision of meat and alternative foods was slightly greater in intervention centres (0·5 (0·3–0·9) serves/d) than comparison centres (0·4 (0·0–0·7) serves/d).

**Table 3:** Daily food group provision and intake to 2–5-year-old children at follow-up as assessed by plate waste in serves (*n* 224)*

Food group	Target serves†	Comparison group (*n* 126)				Intervention group (*n* 98)			
		Serves provided		Serves consumed		Serves provided		Serves consumed	
		Median	IQR	Median	IQR	Median	IQR	Median	IQR
Vegetable	1–1·5	0·8	0·5–1·3	0·5	0·3–0·9	0·9	0·7–1·2	0·5	0·2–0·8
Beans and legumes‡	–	0·0	0·0–0·0	0·0	0·0–0·0	0·0	0·0–0·0	0·0	0·0–0·0
Fruit	1	1·5	0·7–2·4	1·0	0·4–2·1	0·6	0·5–0·9	0·4	0·2–0·6
Cereals and breads	2	2·0	1·5–2·9	1·6	1·0–2·4	1·2	0·9–2·0	0·9	0·5–1·5
Dairy and alternatives	2	0·7	0·0–1·8	0·6	0·0–1·5	0·9	0·5–1·4	0·7	0·1–1·0
Meat and alternatives	1	0·4	0·0–0·7	0·2	0·0–0·5	0·5	0·3–0·9	0·2	0·1–0·6
Unsaturated fats and oils	0–1	0·7	0·2–2·1	0·6	0·2–1·7	0·5	0·2–0·6	0·3	0·1–0·5
Discretionary	0	0·2	0·0–0·6	0·1	0·0–0·4	0·1	0·0–0·1	0·0	0·0–0·1

IQR, inter-quartile range.

* Data presented as median (IQR).

† Targets using Victorian Menu planning guidelines for long day care^(17)^.

‡ Fits within the vegetable, and meat and alternatives groups, no specific target.

**Table 4:** Impact of intervention on differences in child food group provision and consumption in serves at follow-up, linear mixed model outputs, in children present at follow-up (*n* 224)

Child provision			Child consumption		
Food group	Estimate	95 % CI	Food group	Estimate	95 % CI
Vegetable and legumes	–0·2	–1·0, 0·7	Vegetable and legumes*	0·7	0·2, 2·1
Fruit*	0·5	0·1, 1·7	Fruit*	0·4	0·1, 1·4
Cereals and breads*	0·7	0·4, 1·5	Cereals and breads*	0·5	0·1, 2·0
Dairy and alternatives	1·1	–2·8, 5·0	Dairy and alternatives	–0·4	–3·0, 2·3
Meat and alternatives	0·1	–0·6, 0·8	Meat and alternatives	0·0	–0·6, 0·6
Unsaturated fats and oils*	0·7	0·1, 3·9	Unsaturated fats and oils*	0·4	0·1, 2·8
Discretionary*	0·5	0·0, 9·2	Discretionary*,†	0·3	0·0, 9·1

* Log-transformed data, exponentiated coefficients reported (ratio of geometric means).

† Heteroscedasticity present in model.

### Menu compliance

At follow-up, half of the intervention centres (*n* 2) were compliant with menu planning guidelines for all food groups, compared with no comparison centres. The remaining two (out of 4) intervention centres were compliant with four out of five food groups (not compliant with dairy and alternative food group; Table 5). Intervention centre menus provided 2·0 ± 0·7 mean serves of vegetables and legumes at follow-up, exceeding the recommended target (1–1·5 serves) by 0·5–1 serve. Similarly, mean serves of meat and alternatives exceeded the recommended target (1 serve) by 0·3 serves for intervention centres.

**Table 5:** Number of centres meeting or exceeding menu planning guidelines at baseline and follow-up (*n* 8)

Food group	Target serves*	Comparison group (*n* 4)				Intervention group (*n* 4)			
		Baseline		Follow-up		Baseline		Follow-up	
		*n*		*n*		*n*		*n*	
Number of centres meeting or exceeding guidelines									
Compliant for all food groups (5/5)	–	0		0		0		2	
Compliance with:									
Vegetable and legumes	1–1·5	1		1		0		4	
Fruit	1	4		4		4		4	
Cereals and breads	2	4		4		3		4	
Dairy and alternatives	2	1		2		2		2	
Meat and alternatives	1	1		0		0		4	

		Mean	sd	Mean	sd	Mean	sd	Mean	sd
Mean daily servings on menu									
Vegetable and legumes	1–1·5	0·9	0·3	1·0	0·3	0·8	0·2	2·0	0·7
Fruit	1	1·7	0·3	1·6	0·5	1·8	0·7	1·1	0·1
Cereals and breads	2	2·5	0·6	2·3	0·3	2·0	0·4	2·2	0·2
Dairy and alternatives	2	1·7	0·3	2·0	0·4	1·9	0·3	1·9	0·1
Meat and alternatives	1	0·7	0·2	0·6	0·0	0·8	0·1	1·3	0·2

* Targets using Victorian Menu planning guidelines for long day care^(17)^.

### Staff acceptability

Overall, all intervention centre directors (four out of four) agreed that they would continue to use the menu box delivery service if able to, whereas only half of the cooks (two out of four) agreed (Table 1). Within comparison centres, one director (out of four) and two cooks (out of four) agreed that they would continue to use the online cook training or would recommend it to other centres. One cook (out of four) and one director (out of four) agreed that they would continue to use the menu assessment tool to plan menus at their centre.

### Fidelity

All intervention centre cooks (4/4) reported using the menu box delivery service and recipes over the 8-week intervention period. Intervention centre cooks reported modifying recipes to speed up preparation times and serving meals to children in ways they believed to be more preferable to children. For example, pasta and pasta sauce are being served separately. Adherence to and use of the online cook training and menu assessment tool was low among cooks from comparison centres. Three (out of four) completed the training, but no cook reported assessing more than one of the 4-week menus using the menu assessment tool.

### Cost

In total, 59/64 invoices were collected from centres over the 8-week intervention period. Five invoices (8 %) were missing across two comparison centres. Missing invoices were assumed to be non-biased and missing completely at random as missingness resulted from cooks misplacing invoices. Mean intervention centre menu expenditure was AUD$4·62 (95 % CI ($4·58, $4·67)) per child/d, compared with AUD$2·28 (95 % CI ($2·27, $2·30)) per child/d in comparison centres.

## Discussion

This study evaluated the impact of a menu box delivery service to support menu compliance and improve food provision and children’s dietary intakes in the ECEC setting. While intervention centre menus showed greater compliance with food group guidelines at follow-up, the impact observed was similar between the intervention and comparison centres in regard to child food provision and intake, including vegetable intake. Satisfaction and acceptability of the intervention were low for cooks but greater for directors, albeit pilot process data (*n* 8). Findings of this study suggest that the menu box delivery service is a viable food service model for the LDC setting and shows improvements in menu compliance with guidelines, delivering child mealtime food provision and intakes similar to those of centres assigned standard practice (comparison), at a greater cost than standard practice.

Evaluations in the literature of childcare centre menu compliance with Australian menu guidelines demonstrate that the provision of vegetables and legumes, as well as meat and alternatives, is consistently least likely to meet menu planning guidelines of all food groups^(10,40,41)^. In our study, mean serves of vegetables and legumes and meat and alternative foods on the menu increased in all intervention centres. These improvements are similar to those seen in past interventions and pre-post studies that aimed to improve menu compliance within the ECEC^(42,43)^. Childcare intake and menu compliance literature illustrate that centres often meet guidelines for centre-level provision of both fruit and cereals and breads and in some cases trend towards over-provision, typically due to the low cost and high palatability of these foods^(27,44)^. In this study, mean serves of fruit and cereals and breads, on menus in intervention centres, were lower than that of comparison centres at follow-up. However, over-provision of both fruits and cereals and breads food groups may lead to displacement of other important food groups, such as vegetables and meats^(27)^. Increases in menu compliance in the menu box delivery service group are consistent with the findings from previous Australian menu planning studies^(34,42)^.

Importantly, however, the impact of the menu box delivery service intervention on food provision and child intake was equivalent to, but not superior to comparison centres following usual practice. These findings are similar to those reported in previous studies. For example, Bell *et al.* (2015) evaluated the *Start Right–Eat Right* program in South Australian LDC centres and found that despite centres increasing the serves of vegetables on their menu to meet menu guidelines, children consumed approximately 50 % of recommendations^(42)^. Similarly, although Grady *et al.* (2020) reported a mean 2·04 ± 0·97 serves of vegetables at 12-month follow-up of a web-based menu planning intervention, child-level outcomes reported in a sub-sample found consumption remained below recommendations, at 0·73 ± 0·72 serves/child^(45)^. Together, these findings suggest that improving menu compliance alone may not be sufficient to improve child food provision and consumption. As educators are responsible for serving food to children at mealtimes, there is a need to understand how educator knowledge and attitudes could impact serve sizes provided to children at mealtimes and how these align with guidelines.

Previous literature, both in Australia and internationally, has found that effective interventions to promote healthy eating in children within the age group attending ECEC settings (2–5 years) are those that target both environmental- and individual-level factors through multicomponent interventions^(10)^. That is, interventions with the greatest impact on child-level outcomes, such as child dietary intake and weight status/BMI (kg/m^2^), have focussed on environmental changes including menu modifications and food policy within multicomponent interventions^(10)^. As the menu box delivery service has the potential to improve the centre food environment through alignment with menu compliance guidelines, combining it with a child-centred intervention, such as vegetable-focussed curriculum activities, could be an effective approach to improving child consumption^(10)^.

Despite acceptability of the menu box delivery service intervention being low for cooks (similar to that for cooks in the comparison group), director acceptability was high amongst pilot questionnaire data with intervention centre cooks and directors (*n* 8). That is, all intervention centre directors agreed that they would use the menu box delivery service again, that children benefitted from the service and that they would recommend the service to other centres. Feedback regarding the menu box delivery service (from intervention centre cooks) and the online cook training and menu assessment tool (from comparison centre cooks) indicated lower agreement with positive statements among cooks than in other studies. For example, in the evaluation by Grady *et al*. (2020) of a web-based menu planning intervention in 25 LDC centres in Adelaide, Australia, found supervisors’ responses showed high agreement with statements related to the acceptability of the tool (88 %)^(45)^. As the main user of the menu box delivery service, cook acceptability is essential, with findings suggesting improvements might be required to the service to increase acceptability prior to larger scale roll-out.

Food budget restrictions can play a role in centre-level food provision decisions in childcare centres, particularly related to types or quality of foods provided^(46)^. Menu budgets for LDC centres vary between individual centres as well as from state to state and country to country and are not regulated. For example, in Western Australia, food budgets can range from AUD$1·17 to AUD$4·03 with an average of approximately AUD$2·00 per child/d^(27)^. In contrast, centres in New Zealand had a median menu expenditure per child/d of NZ$3·68 (mean $5·06 ±$3·09; range $0·90–16·00)^(21)^. In this study, mean menu expenditure, per child/d, of intervention centres was AUD$2·34 greater than expenditure in comparison centres. Increases in menu expenditure may allow centres to improve menu compliance. Sambell *et al.* (2020) found an increase in average food expenditure of AUD$0·50 per child/d may significantly improve menu compliance; however, implications on practice require further exploration^(27)^.

Strengths of this study include the novelty of the intervention being the first study to explore a menu box delivery concept outside the commercial household environment, the cluster randomised controlled study design and staff (director and cook) feedback. However, a number of limitations exist. First, the 8-week intervention implementation period may not have been an adequate duration to observe changes in children’s dietary intake given the variability in day-to-day child attendance in care. Healthy eating interventions in child care are recommended to have a minimum 12-month duration, or ideally 2 to 4 years^(10)^. Although individual child-level plate waste was measured to estimate children’s food consumption (a valid and comprehensive dietary intake assessment method), centre-level food provision and waste were not measured due to study time constraints. As children’s meals were served by educators in the room, it is unclear how much food was provided to each room as a whole and consequently how much was not served to children. While a sample size of >200 children was achieved (more than the sample size estimation required), the number of cooks and centres involved in the study was limited to four in each group. This is a smaller sample size than other studies in the LDC setting that have collected cook or director feedback through questionnaires or interviews^(15,24,26,47)^. Future trials should focus on achieving a larger sample size of cooks and undertake more in-depth qualitative interviews, to achieve richer feedback should focus on achieving a larger sample size of cooks and undertake more in-depth qualitative interviews, to achieve richer feedback on achieving a larger sample size of cooks and undertake more in-depth qualitative interviews and to achieve richer feedback. Furthermore, involving LDC cooks in the menu box intervention design, utilising co-design methodology may assist in refining the existing menu box delivery service to meet the specific needs of the industry. Furthermore, intervention centre cooks (7·1 years) were more experienced than comparison centre cooks (3·9 years), which may have impacted their skills and knowledge in this sector.

Finally, the methodology used for measuring plate waste differed between baseline and follow-up, due to the implications of COVID-19 restrictions. This affected the capacity to draw a direct comparison between baseline and follow-up data. The methodology used to analyse child food provision and consumption follow-up data may have also impacted the reliability of the plate waste measures, as both child dietary provision and consumption were estimated from photographs. The findings of this novel study, which piloted the application of a menu box delivery service in the LDC setting, suggest that this is a viable model. Study findings showed that although menu compliance can be improved via a menu box delivery service, impacts on food provision and consumption in children were similar for the intervention and comparison groups. The innovative combination of sector guidelines and an emerging food service model could support longer-term, sustainable improvements in centre menu compliance, which may improve children’s food provision and intakes in the longer term. Following refinement of the menu box delivery service to improve cook acceptability, future trials are recommended to be conducted in larger samples of cooks to ensure in-depth staff feedback and with longer follow-up periods to determine the long-term effect on child dietary intake. Future opportunities should seek to continue to harness benefits of a meal kit subscription services such as its popularity and convenience^(28,30)^. Additionally, strategies to reduce cost of a menu box delivery service should be employed to ensure the feasibility of adoption of the service into usual practice through establishing economies of scales and strategies such as utilising lower-cost produce (second grade)^(48)^. Furthermore, opportunities should also include exploring the integration of the menu box delivery service into multicomponent interventions that address both environmental and individual-level factors, for example in combination with a child-centred curriculum and/or mealtime environment training for educators.
